# The New Hypnotic—Somnal

**Published:** 1891-03

**Authors:** 


### The New Hypnotic—Somnal.

The ideal hypnotic has not yet been discovered. Chloral hydrate is a great favorite, especially among our asylum superintendents, but it has its manifest drawbacks in private practice. Chloralurethane and chloralamide are competitors for therapeutical favor, along with somnal, all of them depending largely on chloral for their efficiency. This suggests that we may, before long, get some composition on that base of chloral which will be more prompt and manageable than any we now have. Sulphonal has lost some of its hold, by reason of its irregularity, by putting the patient to sleep twelve hours or more after the time when the somnolence was sought, or prolonging it beyond the point desired; its potency in a certain range of cases should not be denied, but taken as a whole the drug has been a disappointment to many. The latest hypnotic, somnal, to receive attention, is preferred by Dr. Kny, the eminent specialist of Strassburg, chiefly on the ground that it has very little influence over the heart's action. The short time, a half-hour or less in many cases, wherein sleep is induced makes somnal much more preferable to sulphonal, which seldom acts under one or two hours, or it may begin to act when the practitioner is about to administer a second dose. The inventor of somnal, Herr Radlauer, of Berlin, has been able to prove that it is a distinct chemical compound, having the formula $C_7H_{12}Cl_3O_3N$. It is obtained from chloral, alcohol, and urethane, and comes as a clear fluid. It differs from chloralurethane in that it contains two atoms more of carbon and four atoms of hydrogen. Radlauer advises that the initial dose shall be 2 grammes, or $\frac{1}{2}$ drachm, mixed either with syrup of raspberry or juice of licorice. The somnolence lasts seven, eight, and even as long as ten hours. Doses of twice the size above mentioned have been given by Prof. Langenbuch, of the Lazarus Hospital at Berlin, without noticing any toxic effects, while the hypnotic result in many cases has been excellent. The quality of sleep is said not to be profound, so that the surroundings should be made as little disturbing as possible.—*Journal of the American Medical Association.*

